# Cases, Illustrating the Employment of Venæsection and Mercury in Angina Trachealis, or Croup

**Published:** 1817-06

**Authors:** Shirley Palmer


### *Cases, illustrating the Employment of Venæsection and Mercury in Angina Trachealis, or Croup.*

By Shirley Palmer, M. D.

By one of my most highly-valued Correspondents, in a distant quarter of the island, I have been requested to state my opinions upon the treatment of Croup, and the results of my experience in it. The former, I believe, have nothing novel or peculiar to recommend them. The latter, truth compels me to declare, is very limited; as the district to which my practice has hitherto been confined, is rarely visited by the ravages of this terrible disease. Within the last ten years, six cases of Croup only have fallen under my observation.

What I have seen of this malady, or experimentally know respecting its treatment, little as it is, I feel pleasure in communicating. To the liberal mind, the diffusion of whatever knowledge it may possess, is scarcely less gratifying than the acquirement.

Of the six cases which I have observed, three only will require to be minutely recorded. The others, slight in character, and treated immediately on the occurrence of the symptoms, by the usual evacuants, by blistering, and the warm bath, yielded with facility. Among the histories which I shall present, the first will be found interesting from its complication with measles; and the second, from the success of powerful depletion, when the second stage of the disease was very far advanced, and the situation of the child apparently desperate. The third, wherein the fatal event was probably accelerated by my solicitude to afford relief, I particularly recommend to the earnest attention of my younger and more inexperienced readers. To them, and indeed to practitioners of every age and description, it is calculated to convey a lesson, salutary as impressive.

CASE 1. On Tuesday evening, December 26th, 1815, while in attendance upon a dangerous case at a village distant but a few miles from this place, I was requested by a respectable farmer to visit one of his children, a thin, delicate boy, aged about seven years He had been indisposed with a cold for two or three days; and, on that morning, was first perceived to "breathe very oddly:" in other respects, nothing formidable was apprehended. Measles, at this time, was prevalent in the village; and the boy, having been much exposed to the infection, was supposed to be sickening of the disease. On ascending the steps which led to the house, the distinct and well-known sound of croupy respiration attracted my attention, and at once announced the nature of the enemy with which we had to contend. It was one of the most severe and strongly-marked cases of croup I have ever seen. The respiratory functions were executed with considerable difficulty; the pulse much accelerated; the temperature of the trunk greatly above, while that of the inferior extremities was below, the natural standard. At the same time, the eyes and nostrils exhibited the ordinary signs of an impending catarrhal affection, and the whole countenance of the child wore an air of indifference which I never before remember to have noticed in so young a subject. No time was to be lost. The lower extremities were immediately immersed, knee-deep, in hot water;* six grains of submuriate of quicksilver with

* It has frequently been a source of wonder and regret to me that, considering the great relief which may be obtained from warm pediluvia, properly employed, in many acute and dangerous diseases, no apparatus has been yet invented from which the full effect of this simple and beneone grain of tartrite of antimony, procured from the medicine-chest of a neighbouring gentleman, as soon as possible administered; and four leeches applied to the superior part of the sternum. A messenger also was dispatched to recall Mr. Bennet, surgeon of this place, who had recently quitted me on a visit to a village not far distant. Half an hour had scarcely elapsed when this gentleman arrived, and, at my request, drew between four and five ounces of blood from the arm of the child in a full stream, while the legs yet continued immersed in the warm water. The leeches, meantime, had performed their office well; and copious vomiting had been twice or thrice induced by the antimonial; and by the conjoint operation of these remedies, respiration was conspicuously relieved; and the impetus of circulation, and the heat of the surface, very satisfactorily lowered. The child now complained of a sensation of faintness. He was, therefore, put to bed: and my directions were that the atmosphere of his chamber should be kept, nearly as possible, of a comfortable and regular temperature: that liquids only should be administered, and of them nothing stronger than tea or barley-water; that four grains of submuriate of quicksilver should be given, with half an ounce of almond mixture, every three hours, and a large blister, sent for the purpose, applied across the throat in the event of respiration becoming again difficult. Considering it, however, highly probable that, under all circumstances, measles might declare itself on the decline of the tracheal symptoms, I requested the relatives of Master C. to immediately discontinue the exhibition of

---

ficent operation may be derived. It is, by no means, sufficient to plunge the extremities of the patient, ankle-deep, for a few minutes, into water contained in an open vessel, as is most commonly done. To obtain all the advantages which the process is capable of yielding, the whole of the legs should be immersed during forty minutes or an hour; the original temperature of the bath kept up by occasional abstraction of the contained, and cautious addition of fresh water; and access of the external air prevented. All these objects might be fully obtained by the employment of a tin bath, of sufficient capacity and depth, furnished with a cover, the apertures of which were so contrived as to closely embrace limbs of any circumference; with an external funnel, fitted to the orifice of a finely perforated spiral tube, by which hot water might be supplied without disturbing the patient, or inconveniently raising the temperature of the bath at any one part; and, lastly, with a stop-cock inserted near the bottom, by which the superfluous fluid might be drawn off. They, who have experienced the relief which often results from pediluvia, effectually employed, in some of the most distressing affections of the chest, will not consider these hints wholly destitute of interest.

the calomel powder on the slightest appearance of any cutaneous eruption, and send off a messenger to inform me of the event.

On repeating my visit the next day, scarcely a trace of the croupy symptoms was perceptible; but respiration, though neither noisy nor difficult, was yet considerably accelerated. The child was feeble, querulous, constantly restless. The bowels had been freely evacuated; and their discharges were most unnatural and offensive. There was occasional slight cough. The curative plan was continued without alteration.

No particular alteration was observed in the symptoms, or directed in the treatment, on the twenty-eighth. The child continued feeble, fretful, and restless. The croupy symptoms had not recurred.

In the afternoon of the twenty-ninth, an eruption of measles on the face was distinctly visible. The child had now lost much of his former irritability and oppression. The frequency of respiration was greatly reduced. The intestinal discharges yet retained their unfavourable appearance; and, as I considered it an object of no mean importance to correct these without producing a too powerful operation on the bowels during the developement of the cutaneous affection, the more active mercurial preparation was discontinued; and a powder, composed of quicksilver with chalk and tartrite of antimony, directed to be administered every six hours. In other respects, the original plan was observed.

Towards the evening of the thirty-first, the cutaneous eruption, which had shewn itself in a very vigorous form, declined somewhat abruptly, and the chest became formidably affected. Master C. passed a disturbed and sleepless night. On the morning of January the first, I found him in a very unpromising state. Respiration was short and difficult; the cough painful and harassing. Some mucus, tinged with blood, had been expectorated during the night. The pulse was thready, rapid, scarcely perceptible; the extremities cold; the tongue covered with a brownish fur. An exhausting diarrhœa had commenced. Frequent immersion of the lower limbs in hot water, and the unremitting application of warmth to them, was now prescribed; coffee, egg, animal broth and jellies, to be employed as food: and half an ounce of an infusion of conserve of roses, gratefully acidulated with muriatic acid, to be administered every three hours. The region of the sternum was, at the same time, directed to be well rubbed, night and morning, with camphorated antimonial ointment. Under this plan, the pulmonary symptoms were speedily relieved by a pustular eruption on the chest. The strength and appetite of our little patient began slowly and progressively to revive. By the assistance of an occasional purgative, the functions of the intestinal canal were restored to their natural state; and on January the 5th, Master C. though still weak, was so far recovered as not to stand in need of my farther attendance.*

No doubt exists in my mind, that Master C. had been suffering the precursory symptoms of measles, for a day or two previously to the evening on which I first visited him; that the inflammation of the nasal and faucial membrane, which ushers in the eruption, aggravated by unrestricted exposure to an intensely cold atmosphere, had been diffused to that portion of the membrane lining the larynx and trachea; and hence all the phænomena of tracheitis, or acute croup, had been suddenly developed. During that unusually severe season, the boy had run about the farm-yard as usual, until rendered by increasing illness unable longer to sustain the exertion.

This, I may be allowed to observe, is the only case of measles, complicated with croup, which has yet fallen under my notice; nor do I, at this moment, recollect that such complication is mentioned in any medical work, except one recently published on the Continent.† What is there advanced upon the subject, scarcely possesses sufficient importance to merit transcription.

Case 2. About two years ago, a child was brought for advice to a professional gentleman in Staffordshire, at whose house I then chanced to be. The respiration indi-

* On the evening of January 6th, a messenger came to inform me, that a younger sister of Master C. aged about three years, had that day been seized with symptoms of croup. I recommended precisely the same remedies to be employed under the superintendance of Mr. Bennet, as had proved so successful in the brother's case; and requested to be again summoned if my attendance should be deemed necessary. I, however, heard nothing farther at that time; but Mr. Bennet has subsequently informed me, that the subsidence of the croupy symptoms was succeeded by the eruption of measles exactly as in the other case; and that the latter terminated altogether very favourably. In a note, with which this gentleman has to day favoured me, on the subject, he states "that the little girl was treated precisely in the same manner as Master C."

† *Dictionnaire des Sciences Médicales.* Tome VII. Article, *Croup.*

cated the existence of severe croup; and I was requested by my friend to see the case. The subject was a boy, aged four years, stoutly built and heretofore invariably healthy. His breathing was dreadfully embarrassed; his eyes watery and dull; his lips and ears almost purple; his cheeks, of a leaden colour; the superficial veins of the head and neck, excessively loaded; the carotid arteries, throbbing with unusual force; the extremities cold; the pulsations of the artery at the wrist, small, feeble, and innumerably rapid. The poor little fellow could only support himself on his legs for a few minutes by reclining against his mother's knee. From her, we unfortunately could gain no precise information respecting the origin and progress of the disease. The child had been very unwell, at times, for nearly a week. During the last two days, he had been growing constantly worse, and had experienced no perceptible interval of relief. His respiration, on the preceding evening and throughout the night, had been so difficult as to induce an apprehension in those around, that "every breath would be the last." The appetite had been very bad; the bowels not so open as usual. No medicine had been administered. As the first and active stage of the disease was evidently gone by, and the signs of exhaustion were very conspicuously marked, I hesitated long respecting the expedience of general abstraction of blood from the system. Something, however, must, without loss of time, be attempted to relieve the oppressed respiration, or the child would inevitably perish. The application of leeches I should certainly have preferred; but some hours must have elapsed ere these could be procured: and knowing the indifference which poverty commonly begets, I felt very doubtful whether the parents of the child would make the efforts and sacrifices necessary to procure them. The external jugular vein rose unusually prominent beneath my finger. I requested the surgeon to plunge his lancet into it. The gush was more impetuous than I ever have seen it from the section of a vein. In less than a minute, nearly eight ounces of blood had been lost; and the child sank, apparently lifeless, on his mother's lap. A striking alteration in the character of the countenance and respiration was observed on recovery from a somewhat alarming state of syncope. The lips had lost their purple hue; the cheeks were completely bleached; the superficial veins had disappeared; and the respiratory process was executed with an almost natural freedom and tranquillity. Four grains of submuriate of quicksilver were directed to be taken every four hours; frequent warm pediluvia, and the necessary restrictions with respect to diet and temperature, enjoined. For many weeks afterwards, I had no opportunity of making any inquiry concerning the issue of the case. The impression on my mind was, that it must have been almost certainly fatal. My pleasure, however, was great on at last learning from my friend, that the mercury had been adequate to effect the revolution in the system of our little patient which the blood-letting had prepared. The croupy symptoms recurred, but not in their original intensity, some hours after the venæsection. No sooner had a large quantity of dark and offensive fæces been evacuated by the operation of the calomel on the intestinal canal, than they finally disappeared: and the child speedily regained his pristine health and vigour.

This was daring practice; and such certainly as the urgent character of the symptoms, and peculiar circumstances of the case, could alone justify. Had the means of local abstraction of blood been within our reach, I should not have ventured, in this advanced stage of the malady, to propose venæsection: nor, indeed, was it my intention that so much of the vital fluid should have been lost; but from the unusual size and impetuosity of the stream in which it rushed from only a moderate orifice of the vein, the surgeon was somewhat taken by surprise. The plan of treatment, which, I conceive, might be more correctly and generally applicable to the situation of a croupy patient, thus perilous and almost desperate, will be cursorily specified in the few observations which the retrospect of my last and fatal case is calculated to suggest.

Case 3. A thin, pale, delicate looking girl, of seven years, had been very unwell for three days, in consequence, it was imagined, of repeated exposure to wet and cold. The family, wholly unacquainted with the signs, and probably even the name, of croup, never once apprehended the existence of danger. Accidentally passing the house, one evening in the spring of 1811, I was desired to see her. The phænomena of tracheal inflammation were too distinctly marked to require deliberation in fixing the character of the disease, or justify a want of promptitude and decision in practice. The second stage was evidently far advanced. Breathing was performed with an almost convulsive effort; and suffocation seemed to be impending. The face had a livid appearance; but the lips were less purpled; the muscular forces less broken; the extremities warmer, and the pulse more firm and distinct, than, from the general aspect of the child, I conld have imagined. She uttered no complaint, and scarcely spoke, except when impelled by urgent thirst to call for water. She was constantly restless, and incapable of remaining longer than a minute or two in a sitting or recumbent posture. I shall not readily forget the expression of deep but silent distress marked upon her countenance as she stood, when I first entered, with her arms folded across, gazing steadfastly on the window, and apparently inconscious of every thing around her. The bowels had been open that day; but of the nature of the discharges, nothing was known.

No leeches could be procured in the neighbourhood. The nearest town was some miles distant. This information immediately turned the scale in favour of venæsection—a remedy in determining the propriety of which my mind had been, for some minutes, most anxiously employed; and which I had resolved only to have recourse to, in the event of my enquiry for leeches proving unsuccessful. Four ounces of blood were taken in a moderate stream from the arm. It had an unusually dark appearance. The respiration of the child was instantly relieved; but she complained of extreme faintness; and the signs of exhaustion became, all at once, too strongly marked to be contemplated without alarm. A light cordial was now given; and the lower extremities plunged into warm water, apparently with good effect: for the child gradually revived: though she yet evinced symptoms of unusual languor, and the action of the heart remained exceedingly depressed. After administering a dose of the mercurial submuriate and tartrite of antimony, procured from a gentleman in the vicinity, I left the child, in order to visit another patient; and hoped to return ere the operation of the antimonial had ceased. But the intelligence soon reached me, that without betraying any sudden alteration, or expressing the slightest disposition to nausea or vomiting, the poor girl had gradually sunk away, and expired shortly after my departure.

The privilege of anatomical examination was granted; and of this I availed myself on the second day. *Dissection.* The whole canal of the trachea, and its primary ramifications, were plugged with a substance not like the firm adherent membrane usually found on inspection of croupy subjects, but very strongly resembling pale honey.

The mucous membrane, from the bronchiæ to the larynx, exhibited decided traces of active inflammation. The bronchial tubes lower down poured out, upon incision, a great quantity of ropy mucus. The blood-vessels of the lungs were loaded with very dark blood. There were some pleuritic adhesions. The pulmonary cavities of the heart were gorged with black loose coagula; the aortic empty.* A large collection of fæcal matter was lodged in the colon. Marks of increased action were visible in several parts of the intestinal tube.

To all who attentively peruse this last history, it will, I think, be apparent, that the death of the child was accelerated by the loss of blood. At the same time, it is very questionable whether, in the desperate circumstances under which I first saw the child, any remedial plan which could have been instituted, would have proved eventually successful. Were I, however, again called to a patient far advanced in the second stage of croup, I would not, unless some peculiar feature or unusual complication† strongly indicated its employment, prescribe *general* blood letting. After administering a full dose of submuriate of quicksilver, or of that and the antimonial tartrite combined, to unload the intestinal canal, I would have the inferior extremities, and if possible, the superior also, immersed for half an hour, or an hour, in hot water. Meanwhile, leeches should be applied about the superior margin of the sternum. The contents of the stomach and bowels having been evacuated, I would commence the regular exhibition of the mercurial submuriate conjoined with opium, and followed by the fœtid spirit of ammonia in musk mixture, almond mixture, or other appropriate vehicle. If the symptoms of the case were very urgent, mercurial frictions might prove a valuable auxiliary. The strength of the patient should be supported throughout by frequent ingestion of coffee, egg, broth, animal jellies, and any of the lighter and more nutritive alimentary preparations. This plan must, of course, be regulated in its application to individual cases, by the age and constitutional peculiarities of the patient, and the precise character and cir-

---

* One of the columnæ carneæ of this ventricle was of such an unusual size, and so formed and connected, as apparently to divide it into two distinct cavities.

† Of this complication, the second case presents an example. The vessels of the head were excessively loaded: and to this circumstance, the success of venæsection is, perhaps, in great measure, attributable. I should have mentioned, that the child, previously to the operation, was uncommonly drowsy.

cumstances of the disease. In laying down rules for the treatment of any complaint, it is obviously impossible to do more than trace a general outline which the judgment and discrimination of the practitioner may enable him to modify and adapt to the nature of the case in which its employment shall be indicated. And woe to the patients of that physician or surgeon who does not constantly and rigorously exercise these important faculties, when summoned to the bed of sickness!

With respect to the treatment of croup in its first and inflammatory stage, one opinion only seems now to prevail among the more enlightened of our profession in this island. The efficacy of calomel, employed in the vigorous manner which Dr. James Hamilton* has recommended, after having been for some years subjected to the test of experiment, is very generally admitted and relied upon. Yet, exalted as is the rank which this powerful medicine holds in my estimation, I would by no means adopt or recommend it in severe and genuine croup, to the exclusion of other remedies, particularly general and local abstraction of blood. On this subject, my opinions correspond precisely with those of Mr. John Burns, of Glasgow.

In the preceding paper, I have merely aimed at a succinct exposition of the more prominent facts, respecting croup, which have come under my own eye, and the results of my own limited experience in its treatment. To investigate disputed points of practice in this formidable malady, or review the mass of written opinion concerning it, which medical literature exhibits, has formed no part of my object. So, in replying to a query of my correspondent respecting the best writers upon cronp, British and foreign, I shall not attempt to trace its literary history; but simply enumerate a few of the more valuable works which I have myself read, or the characters of which I have received from persons of correct judgment and of unquestionable authority.

The following short list will, I believe, be found to comprehend every thing of value which has yet appeared upon the symptoms, pathology, and treatment, of the *cynanche trachealis*. The many excellent observations upon it, scattered in the British and continental Journals of medicine, and other periodical works, obviously do

---

* Dr. Hamilton seems to have borrowed the idea of the calomel treatment of croup from the practice of the American physicians.

not come within the pale, to which this concise notice must necessarily be restricted.

*British writers.* Francis Home. Inquiry into the nature, cause, and cure of the croup. 8vo. Edinburgh. 1765. —Thomas Crawford. Disquisitio medica inauguralis de cynanche stridulâ. 8vo. Edinburgh. 1771.—Inserted also in the third volume of the work, entitled Thesaurus medicus, &c. 8vo. Edinburgh and London. 1785.—John Cheyne. Pathology of the membrane of the larynx and bronchia. 8vo. Edinburgh. 1810.—Valuable observations on croup may also be seen in Dr. Johnstone's Treatise on the malignant Angina, &c. 8vo. Worcester. 1779; Dr. Hamilton's Treatise on the management of female complaints. 8vo. Edinburgh. 1809; and Mr. J. Burns' Principles of Midwifery, &c. 8vo. London. 1811.

*French.* Double. Traité du croup. 8vo. Paris. 1811.— Giraudi. De l'angine tracheale, connue sous le nom du croup. 8vo. 1811.—Caillau. Memoire sur le croup. 8vo. Bourdeaux. 1812. — Royer-Collard. Rapport addressé á S. Exc. le ministre de l'interieur, sur les ouvrages envoyés au concours sur le croup, &c. 8vo. Paris. 1812.—This work contains the substance of the two prize essays on croup by M. M. Jurine and Albers; of which a very excellent account is given by Mr. Williams, in the tenth volume of the *New Medical and Physical Journal.* See also Portal's Memoires sur la nature et le traitement de plusieurs maladies, tome troisième; and the Dictionaire des sciences médicales, tome septieme; Article, croup; very elaborately written,

*German.* Murray. Abhandlung von einer boesartigen Brœune, und einer widernaturlischen Haut in der Luftrehre. 8vo. Gœttingen. 1769. There are also some observations on croup, possessing little of originality, in Rosenstein's work, translated from the Swedish by Murray, and entitled, Anweisung zur Kenntniss und fur der Kinderkrankheiten. Gœttingen. 1771

*Italian.* Martino Ghisi. Lettere mediche, la prima delle quali tratta di rari mali, curati col mercurio crudo: e la seconda contiene l'istoria delle angine epidemiche degli anni 1747 e-48. 4to. Cremone. 1749.

